# “Underneath the visible” - COVID-19 Risk prediction tools in a high-volume, low-resource Emergency Department

**DOI:** 10.12669/pjms.39.1.6043

**Published:** 2023

**Authors:** Sama Mukhtar, Sarfaraz Ahmed Khatri, Adeel Khatri, Nida Ghouri, Megan Rybarczyk

**Affiliations:** 1 Sama Mukhtar, Consultant Emergency Medicine, Indus Hospital and Health Network, Karachi; 2 Sarfaraz Ahmed Khatri, Resident Emergency Medicine, FCPS-II Trainee, Indus Hospital and Health Network, Karachi; 3 Adeel Khatri, Consultant Emergency Medicine, Indus Hospital and Health Network, Karachi; 4 Nida Ghouri, Research assistant, Indus hospital and Research Centre, Karachi; 5 Megan Rybarczyk, Consultant Emergency Medicine, Perelman School of Medicine at the University of Pennsylvania, USA

**Keywords:** COVID-19, Mortality, Brescia COVID Respiratory Severity Scale

## Abstract

**Objectives:**

Patient risk stratification is the cornerstone of COVID-19 disease management; that has impacted health systems globally. We evaluated the performance of the Brescia-COVID Respiratory Severity Scale (BCRSS), CALL (Co-morbid, age, Lymphocyte and Lactate dehydrogenase) Score, and World Health Organization (WHO) guidelines in Emergency department (ED) on arrival, as predictors of outcomes; Intensive care unit (ICU) admission and in-hospital mortality.

**Methods:**

A two-month retrospective chart review of 88 adult patients with confirmed COVID-19 pneumonia; requiring emergency management was conducted at ED, Indus Hospital and Health Network (IHHN), Karachi, Pakistan, (April 1 to May 31, 2020). The sensitivity, specificity, receiver operator characteristic curve (ROC) and area under the curve (AUC) for the scores were obtained to assess their predictive capability for outcomes.

**Results:**

The in-hospital mortality rate was 48.9 % with 59.1 % ICU admissions and with a mean age at presentation of 56 ± 13 years. Receiver operator curve for BCRSS depicted good predicting capability for in hospital mortality [AUC 0.81(95% CI 0.71-0.91)] and ICU admission [AUC 0.73(95%CI 0.62-0.83)] amongst all models of risk assessment.

**Conclusion:**

BCRSS depicted better prediction of in-hospital mortality and ICU admission. Prospective studies using this tool are needed to assess its utility in predicting high-risk patients and guide treatment escalation in LMIC’s.

## INTRODUCTION

The unwavering COVID-19 pandemic has affected economies and health infrastructures globally^1^,^2^, especially low and middle-income countries (LMIC’s) like Pakistan.^3^-^5^ The World Health Organization (WHO) surveillance data has reported 599,825,400 confirmed cases and 6,469,458 deaths globally.^6^ Emergency departments (ED) are combating the full spectrum of the disease, as patients’ progress to respiratory failure within 24 hours^7^ with a mortality of 2-3%.^4^ This steep progression poses a challenge in clinical decision-making^6^ and highlights the importance of patient risk stratification, in low-resource ED’S.

Two risk prediction scores developed for COVID-19 are of significance. The Brescia-COVID Respiratory Severity Score (BCRSS) was developed during the COVID-19 outbreak in Brescia, Italy. It is a nine-level (0-8) algorithm that determines patient disease severity at presentation and apportions stepwise treatment recommendation. It requires re-assessment and re-scoring after intervention and tracks the level of respiratory severity.^8^ The CALL (Comorbidity, Age, Lymphocytes, Lactate dehydrogenase [LDH]) score has also been shown to predict disease progression and in-hospital mortality in with; sensitivity 77.25%, specificity 58%, positive predictive value 57.77% and negative predictive value 77.44%.^1^

IHHN is a free of cost, tertiary care center situated in district Korangi, Karachi, Pakistan. The ED provides acute care to more than 400 patients/day. The first case of COVID-19 was reported in Pakistan, on February 26, 2020^9^, and the numbers rose to 1,488,958 by mid- February 2022.^7^ Without clear guidelines and risk prediction tools to effectively allocate resources; the situation was worsened by acute scarcity of ICU beds.^1^,^3^ With the aim of strategic redistribution of hospital resources in a frail health infrastructure;^10^ this study was designed to evaluate the performance of the CALL Score, BCRSS, and WHO guidelines in predicting outcomes i.e. ICU admission and in-hospital mortality. To date, these clinical risk prediction models have not been assessed in our population in the ED.

## METHODS

A retrospective chart review was conducted in the ED, IHHN (April 1 to May 31, 2020). Approval from Institutional review board was obtained under IRD_IRB_2020_06_005 on June 11, 2020. Data was extracted from electronic health management information systems (HMIS), de-identified and coded by a data analyst and entered into a secure electronic database. Data was reviewed by two independent investigators and conflicts were resolved by a third. Data confidentiality was ensured. Patients presenting with pneumonia secondary to other causes were excluded. Patients’ ≥ 14 years of age, with positive Polymerase chain reaction for COVID-19 or high clinical/radiological suspicion, that mandated ED admission on the basis of National Clinical Guidelines^4^ were included. Patients were triaged into (P1: Immediate, P2: very urgent, and P3: urgent) acuity based upon the Manchester triage system (MTS).^11^ The following severity scoring systems were compared.

### WHO guidelines^12^:

Patients were classified into moderate (clinical signs of pneumonia; fever, dyspnea, fast breathing, cough and oxygen saturation SpO2 ≥ 90% on room air [RA]), severe (clinical signs of pneumonia with respiratory rate[R/R] > 30 breaths/ min or SpO_2_ < 90% on RA) and critical (acute respiratory distress, sepsis and septic shock).^10^,^12^

### BCRSS^8^:

Patients were evaluated and allotted one point each for the following variables; wheezing /inability to speak complete sentence at rest, RR >22/min, PaO2 ,65 mmHg or SpO_2_ < 90% in an arterial blood sample, and significant chest X-Ray findings. Scores (0-3) were allotted based at ED arrival.^8^

### CALL Score^13^:

Disease severity ranged from 4 (absence of comorbidity, age ≤ 60 years, lymphocyte count > 1.0 × 10^9^/L, LDH ≤ 250 U/L) to 13 (presence of comorbidity, age >60 years, lymphocyte count ≤ 1.0 × 10^9^/L, LDH > 500 U/L).^13^ The results were assigned into mild (4-6), moderate (7-9), and severe (10-13) at ED arrival. Patients discharged from the hospital were considered recovered. The primary outcome was in-hospital mortality and secondary, ICU admission. 

Data was cleaned and coded prior to analysis on IBM SPSS version 26. Mean ± standard deviation was observed for normally distributed variables with Median with Interquartile range was for skewed data. Normality of data was checked by Shapiro Wilk’s test, histogram, and Q-Q plot. Frequency and percentage were calculated for categorical variables. Association of BCRSS, CALL score and WHO guidelines with outcomes was established by chi-square test. To assess the predictive capability of risk assessment scores for outcome variables; receiver operator characteristic (ROC) curve and area under the curve (AUC), sensitivity and specificity were obtained. Intraclass correlation coefficient (ICC) was derived to evaluate absolute agreement with 95% confidence interval between BCRSS and WHO guidelines at ED arrival. P-Value of ≤ 0.05 was considered significant.

## RESULTS

The records of 167 were reviewed, with a male predominance 129 (77.2%) and mean age of 56 ± 13 years. Non availability of bed resulted in 79(47.3%) patients being referred to other facilities and finally 88 patients were admitted in ED (Table-I). Disease severity on ED arrival of these 88 patients was assessed by WHO guidelines, CALL score and BCRSS and their outcomes were analyzed. Upon comparison, 31(72.1%) patients who died were categorized severe (CALL Score), 25(58.1%) Level-3 (BCRSS) and severe (WHO guidelines) each (Table-II). Of the patients admitted in ICU, 17(47.2%) were categorized in Level-2 and 3 of BCRSS each, while most of the ICU admissions were categorized as severe by WHO guidelines 18(50%). All three models were strongly associated with the outcomes i.e. mortality and ICU admissions. (p-value ≤ 0.05) (Table-II).

**Table-I:** Baseline characteristics of COVID -19 patients, in ED (n=167).

Patient characteristics		Mean ± SD	Median, IQR
Age (years)		56 ± 13	55, 18
Systolic Blood Pressure (mm/Hg)		140.7 ± 22	139, 29
Diastolic Blood Pressure (mm/Hg)		81.7 ± 16.5	80, 20
Heart rate (beats/min)		109.3 ± 22.2	108, 28
Respiratory rate (breaths / min)		31.6 ± 8.5	30, 12
Temperature (°F)		98.4 ± 1.3	98, 0.6
Oxygen saturation (%)		82.7 ± 14.3	88, 19
		n (%)	
Gender	Male	129(77.2)	
	Female	38(22.8)	
Triage acuity	P1	105(62.9)	
	P2	45(26.9)	
	P3	17(10.2)	
Outcome	ICU Admission	36(21.5)	
	HDU Admission	43(25.7)	
	ED death	9(5.4)	
	Referred out	79(47.3)	

**Table-II:** Association of measures of risk with In-hospital mortality and ICU admission of COVID -19 patients, in ED (n= 88).

Risk scores		Outcome		P-value	ICU admission		P-value
	
		Discharged n (%) 45(51.1%)	Died n (%) 43(48.9%)		No n (%) 52(59.1%)	Yes n (%) 36(40.6%)	
BCRSS	L0*	3(6.7)	1(2.3)	<0.001	4(7.7)	0	0.003
	L1^†^	11(24.4)	1(2.3)		12(23.1)	0	
	L2^‡^	27(60)	14(32.6)		24(46.2)	17(47.2)	
	L3^§^	4(8.9)	25(58.1)		12(23.1)	17(47.2)	
	L4^|^	0	1(2.3)		0	1(2.8)	
	L5^¶^	0	1(2.3)		0	1(2.8)	
WHO guidelines	Moderate	36(80)	8(18.6)	<0.001	33(63.5)	11(30.6)	0.01
	Severe	7(15.6)	25(58.1)		14(26.9)	18(50)	
	Critical	2(4.4)	10(23.3)		5(9.6)	7(19.4)	
CALL score	Mild	5(11.1)	3(7)	0.002	7(13.5)	1(2.8)	0.01
	Moderate	24(53.3)	9(20.9)		24(46.2)	9(2.5)	
	Severe	16(35.6)	31(72.1)		21(40.4)	26(72.2)	

* Monitor with pulse oximetry and clinical evaluation. † Provide supplemental oxygen. Monitor with pulse oximetry and clinical evaluation. ‡ Perform Chest-X ray and arterial blood gases. Provide supplemental oxygen and monitor with pulse oximetry and clinical evaluation. § Trial of Non-Invasive ventilation and intubate in case of worsening. |Follow ICU protocol; use local ventilator weaning protocols. ¶ Minimize sedation and daily trial of spontaneous breathing.

WHO guidelines depicted higher sensitivity and specificity as compared to BCRSS and CALL score. The sensitivity was highest (81%) at the diagnosis of moderate disease, with 80% specificity. The best cutoff for CALL score was at moderate disease (sensitivity 72.1% and specificity 64.4%), and in the case of BCRSS the highest cut off was Level-2 (sensitivity 62.8% and specificity 91.1%) (Table-III). For ICU admission, WHO guidelines depicted a sensitivity of 69% and specificity of 63% at moderate disease cutoff level, while CALL score had a sensitivity of 72.2% and specificity of 59.6% for the same. BCRSS had maximum sensitivity (52.8%) and specificity (76.9%) at Level-2 cutoff (Table-III).

**Table-III:** Sensitivity and Specificity of BCRSS, WHO guidelines and CALL score at ED arrival, for predicting In-hospital mortality and ICU Admission.

Risk scores		In-hospital mortality		ICU Admission	

		Sensitivity	Specificity	Sensitivity	Specificity
BCRSS	-1.00	100.000	0.000	100.000	0.000
	Lo	97.700	6.700	100.000	7.700
	L1	95.300	31.100	100.000	30.800
	L2	62.800	91.100	52.800	76.900
	L3	4.700	100.000	5.600	100.000
	L4	2.300	100.000	2.800	100.000
	L5	0.000	100.000	0.000	100.000
WHO guidelines	1.00	100.000	0.000	100.000	0.000
	Moderate	81.400	80.000	69.400	63.600
	Severe	23.300	95.600	19.400	90.400
	Critical	0.000	100.000	0.000	100.000
CALL Score	0.00	100.000	0.000	1.000	0.000
	Mild	93.000	11.100	97.200	13.500
	Moderate	72.100	64.400	72.200	59.600
	Severe	0.000	100.000	0.000	100.000

**Fig.1 (a-b):**
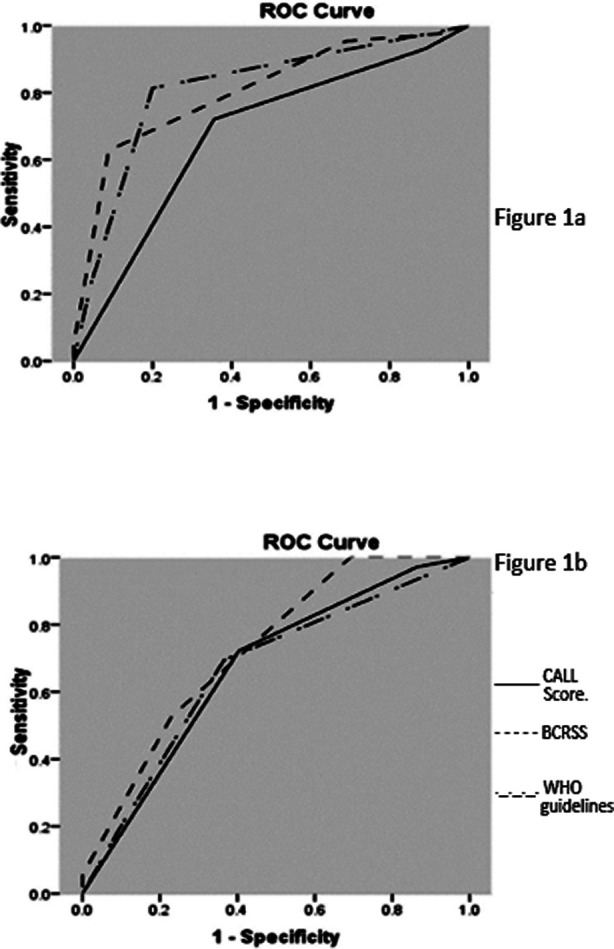
ROC predictive capability of BCRSS, WHO guidelines and CALL Score for; (1a) In-hospital mortality (1b) ICU admission.

BCRSS and WHO guidelines both had good predicting capability for in-hospital mortality [AUC 0.81(95%CI 0.71-0.9)] and [AUC 0.81(95%CI 0.72-0.91)] respectively as compared to CALL score [AUC 0.68(95% CI 0.56-0.71)] (Fig.1a) (Table-IV). BCRSS was most competent in predicting ICU admission among all models of risk assessment (Fig.1b) (Table-IV). A good agreement was documented between BCRSS and WHO guidelines with Cronbach’s alpha 0.81, ICC 0.73 (95%CI 0.34-0.87) (p-value <0.001).

**Table-IV:** AUC for predictive capability of BCRSS, WHO guidelines and CALL Score for In-hospital mortality and ICU admission.

Risk scores	In-hospital mortality				ICU admission			

	AUC	SE	95%CI	P-value	AUC	SE	95%CI	P-value
BCRSS	0.81	0.05	0.71-0.9	<0.001	0.73	0.05	0.62-0.83	<0.001
WHO guidelines	0.81	0.05	0.72-0.91	<0.001	0.67	0.06	0.55-0.78	0.008
CALL Score	0.68	0.06	0.56-0.71	0.005	0.67	0.06	0.56-0.78	0.007

## DISCUSSION

COVID-19 pandemic is a global catastrophe^2^ with in-hospital mortality and ICU admission rate of 32.3% and 31.3% respectively.^14^ Significant mortality rates have been reported in LMICs^3^ and is reaffirmed by Pakistani literature with a positivity rate of 32.4%, ICU admission and in-hospital mortality rate of 59.1% and 48.9% respectively.^3^ This disparity may be attributed to the increased disease burden, scanty resources and delayed presentation in LMIC’s^15^, as evident from increased P1 acuity triages.

There was male predominance with disease prevalence in >50 years of age in congruity with local^1^,^5^,^9^,^16^-^19^ and international literature.^20^ Akin to Nava et al^14^, all strategies for COVID-19 severity evaluation in our study depicted the increasing trend of ICU admission and mortality with worsening risk class. In the current study BCRSS has shown great value in predicting ICU admissions and in-hospital mortality, parallel to results of Rohat et al.^21^, with AUC 0.842 (95% CI 0.799 - 0.884) and 0.804 (95% CI 0.754-0.85) respectively. Similarly, Nava et al.^14^ denoted the superiority of BCRSS in predicting ICU admission.

In our study, CALL Score depicted sub-optimal performance for both outcome variables as also noted in a Peruvian study.^22^ In contrast, Jilanee et al.^1^ reported better sensitivity. This may be explained by lack of inclusion of respiratory parameters, essential for predicting disease progression.^13^

In this study, BCRSS and WHO guidelines performed the best in predicting in-hospital mortality. The ability of BCRSS to identify severe disease earlier may be due to the incorporation of serial examination to guide treatment escalation.^23^ BCRSS scale is new and has shown promising results in international literature.^14^,^21^,^23^,^24^ The efficacy of WHO guidelines remains unexplored in the ED setting; however our findings suggest its potential utility, that mandates further research. This study supports the overall superiority of BCRSS in predicting both ICU admission and deaths, affirmed by Prashant et al.^25^ in India as well. To the best of our knowledge, our work is novel to Pakistan in comparing the predictive capability of risk stratification models in the ED. We hope to extrapolate these results to incorporate BCRSS scale in our low-resource high volume ED, leading to improved patient outcomes.

### Limitation:

Firstly, it is a single-center study with a limited sample size because of the shortage of ICU beds and consequent referrals during the first wave. Though, our positivity, ICU admission and mortality rate were in conformity to national data.^4^ Secondly, cases with incomplete charts were excluded due to retrospective nature of the study.

## CONCLUSION

It is our hope that with this retrospective chart review, we can inculcate BCRSS to augment clinical decision-making and improve outcomes. BCRSS will help identify high-risk patients and guide effective resource apportionment. Though, prospective studies are required in a wider range of settings to gauge its robustness in high-volume, low-resource ED’s.

### Author’s contribution:

**SM & AK:** Article drafting and literature search, accountable for integrity of work. **NG, SM, AK & MR:** Proof reading and result writing. **SM & SAK:** Research conception and design, acquisition of data and interpretation. **NG:** Data analysis and interpretation.
